# Crystal structure and Hirshfeld surface analysis of dimethyl 2-oxo-4-(pyridin-2-yl)-6-(thio­phen-2-yl)cyclo­hex-3-ene-1,3-di­carboxyl­ate

**DOI:** 10.1107/S2056989024004687

**Published:** 2024-05-24

**Authors:** Farid N. Naghiyev, Victor N. Khrustalev, Mehmet Akkurt, Elnur Z. Huseynov, Ajaya Bhattarai, Ali N. Khalilov, İbrahim G. Mamedov

**Affiliations:** a Department of Chemistry, Baku State University, Z. Khalilov str. 23, Az, 1148, Baku, Azerbaijan; b Peoples’ Friendship University of Russia (RUDN University), Miklukho-Maklay St.6, Moscow, 117198, Russian Federation; c N. D. Zelinsky Institute of Organic Chemistry RAS, Leninsky Prosp. 47, Moscow, 119991, Russian Federation; d Department of Physics, Faculty of Sciences, Erciyes University, 38039 Kayseri, Türkiye; e Department of Chemistry, M.M.A.M.C (Tribhuvan University) Biratnagar, Nepal; f "Composite Materials" Scientific Research Center, Azerbaijan State Economic University (UNEC), Murtuza Mukhtarov str. 194, Az 1065, Baku, Azerbaijan; Harvard University, USA

**Keywords:** crystal structure, thio­phene ring, pyridine ring, cyclo­hexene ring, Hirshfeld surface analysis

## Abstract

In the title compound, C_19_H_17_NO_5_S, the cyclo­hexene ring adopts nearly an envelope conformation. In the crystal, mol­ecules are linked by C—H⋯O hydrogen bonds, forming a three-dimensional network. In addition, C—H⋯π inter­actions connect the mol­ecules by forming layers parallel to the (010) plane. According to the Hirshfeld surface analysis, H⋯H (36.9%), O⋯H/H⋯O (31.0%), C⋯H/H⋯C (18.9%) and S⋯H/H⋯S (7.9%) inter­actions are the most significant contributors to the crystal packing.

## Abstract 2

In the crystal, mol­ecules are linked by C—H⋯O hydrogen bonds, forming a three-dimensional network. In addition, C—H⋯π inter­actions connect the mol­ecules by forming layers parallel to the (010) plane.

## 1. Chemical context

The class of mol­ecules known as carbo- and heterocycles, arguably the most important, has a significant impact on the synthesis of various functionalized systems that have found diverse research and commercial applications (Huseynov *et al.*, 2023 ▸; Akkurt *et al.*, 2023 ▸). Bioactive natural and synthetic compounds frequently incorporate carbocycles and heterocycles as fundamental structural components. Moreover, these compounds may play an important role in organic synthesis as starting materials (Maharramov *et al.*, 2022 ▸; Khalilov *et al.*, 2023 **a* ▸,b* ▸). These derivatives have found broad applications in coordination chemistry (Gurbanov *et al.*, 2021 ▸; Mahmoudi *et al.*, 2021 ▸), medicinal chemistry (Askerova, 2022 ▸) and materials chemistry (Velásquez *et al.*, 2019 ▸; Afkhami *et al.*, 2019 ▸). These ring systems are utilized in various applications, spanning pharmaceuticals, ligands, catalysts, materials and beyond (Maharramov *et al.*, 2021 ▸, Sobhi & Faisal, 2023 ▸). Functionalized systems incorporating cyclo­hexa­none, pyridine and thio­phene motifs have demonstrated diverse biological activities, including molluscicidal, anti­cancer, anti­oxidant, cytotoxic, anti-inflammatory, herbicidal, pesticidal, anti­bacterial, and more (Erenler *et al.*, 2022 ▸; Atalay *et al.*, 2022 ▸; Donmez & Turkyılmaz, 2022 ▸). The broad application of these systems has garnered significant attention toward the efficient and regioselective development of such compounds. In summary, the synthesized compound offers a unique combination of structural features, including heteroatom diversity, conjugation, strategic functional group placement, and potential biological relevance. Analysis of its structure and properties can provide valuable contributions to the broader field of carbo- and heterocyclic chemistry and may have implications for various applications, including materials science and medicinal chemistry. Hence, within the context of structural studies (Abdinov *et al.*, 2004 ▸, 2012 ▸, 2014 ▸; Naghiyev *et al.*, 2020 ▸, 2021*a* ▸, 2022 ▸), we present the crystal structure and Hirshfeld surface analysis of the title compound, dimethyl 2-oxo-4-(pyridin-2-yl)-6-(thio­phen-2-yl)cyclo­hex-3-ene-1,3-di­carboxyl­ate. 
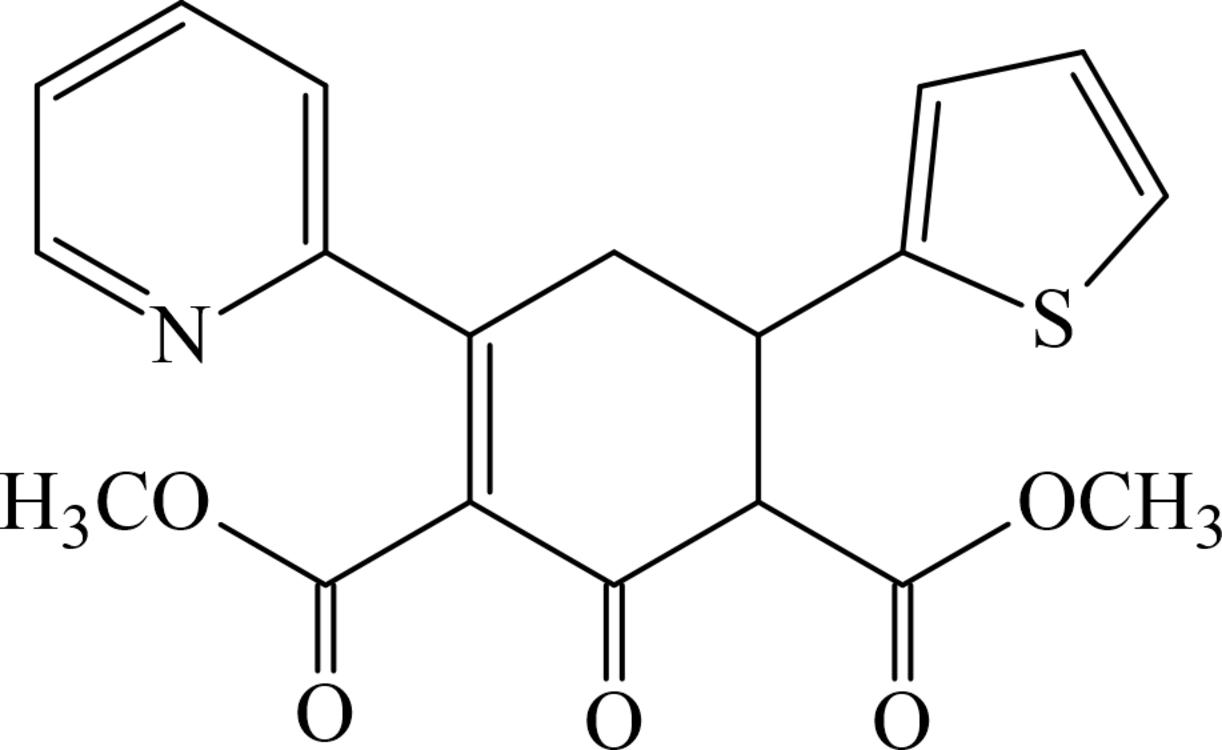


## 2. Structural commentary

**Figure 1:**
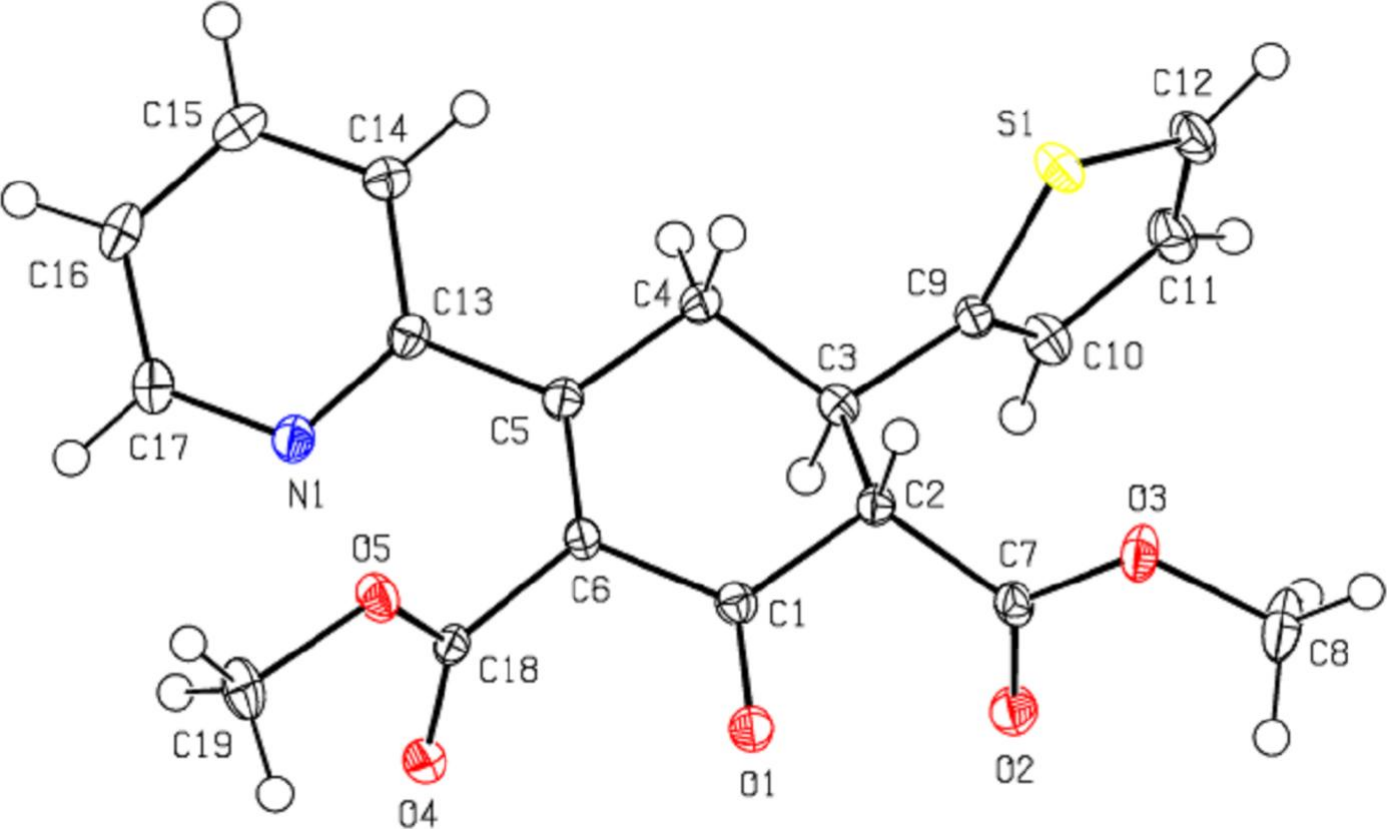
The mol­ecular structure of the title compound, showing the atom labelling and displacement ellipsoids drawn at the 50% probability level.

In the title compound (Fig. 1 ▸), the cyclo­hexene ring (C1–C6) adopts nearly an envelope conformation [puckering parameters (Cremer & Pople, 1975 ▸) are *Q*_T_ = 0.526 (2) Å, θ = 53.9 (2)° and φ = 117.3 (3)°]. The cyclo­hexene ring (r.m.s deviation = 0.002 Å) makes dihedral angles of 84.46 (11) and 29.49 (10)° with the thio­phene (S1/C9–C12) and pyridine (N1/C13–C17) rings, respectively. The angle between the thio­phene and pyridine rings is 77.04 (11)°. The C8—O3—C7—C2, O2—C7—C2—C3, C19—O5—C18—C6 and O4—C18—C6—C5 torsion angles are −169.87 (18), −70.3 (2), 174.97 (15) and 107.7 (2)°, respectively. The geometric properties of the title compound are normal and consistent with those of the related compounds described in the *Database survey* (Section 4).

## 3. Supra­molecular features and Hirshfeld surface analysis

**Figure 2:**
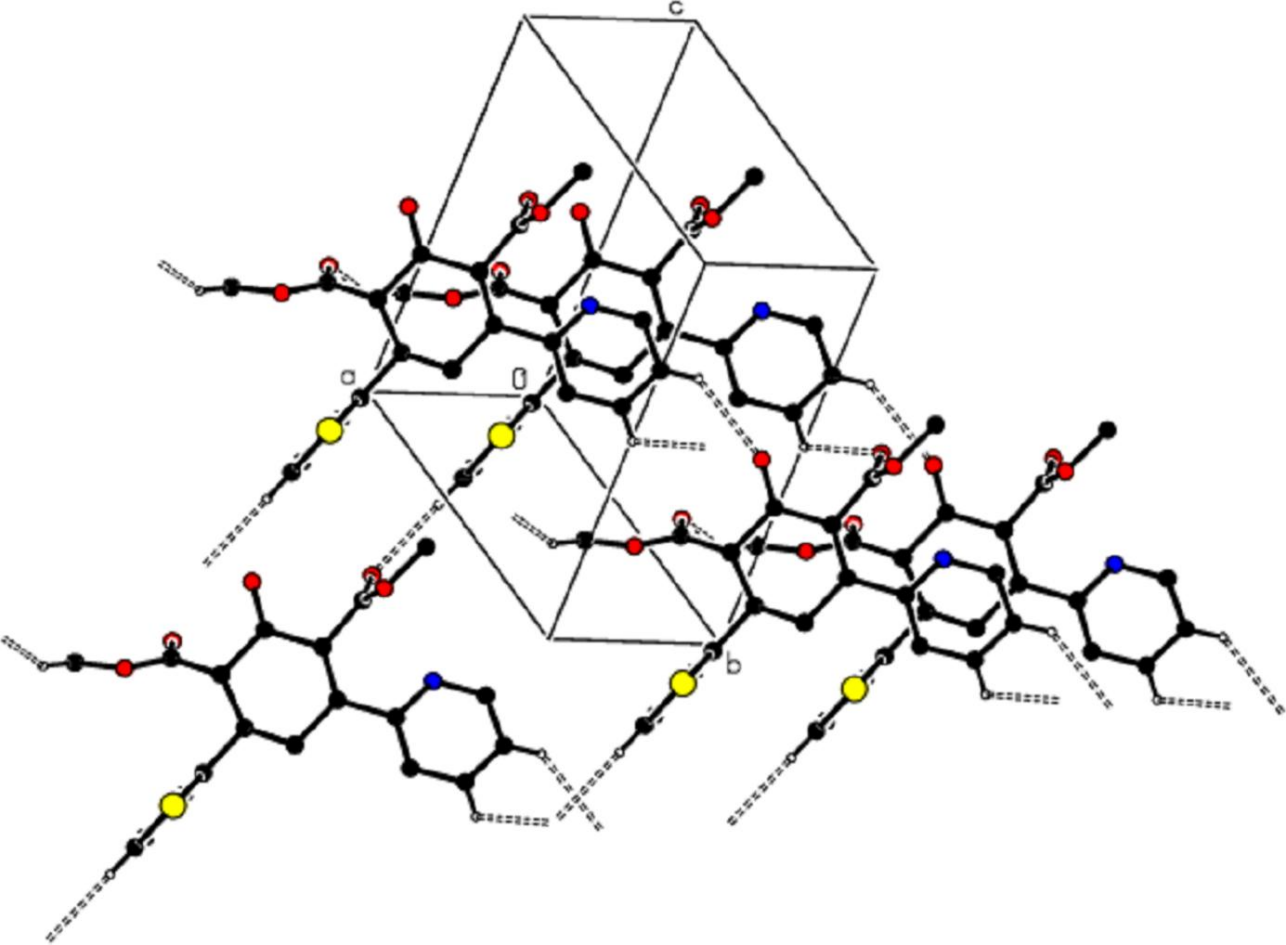
View of partial packing along the *b* axis of the title compound with C—H⋯O hydrogen bonds shown as dashed lines.

**Figure 4:**
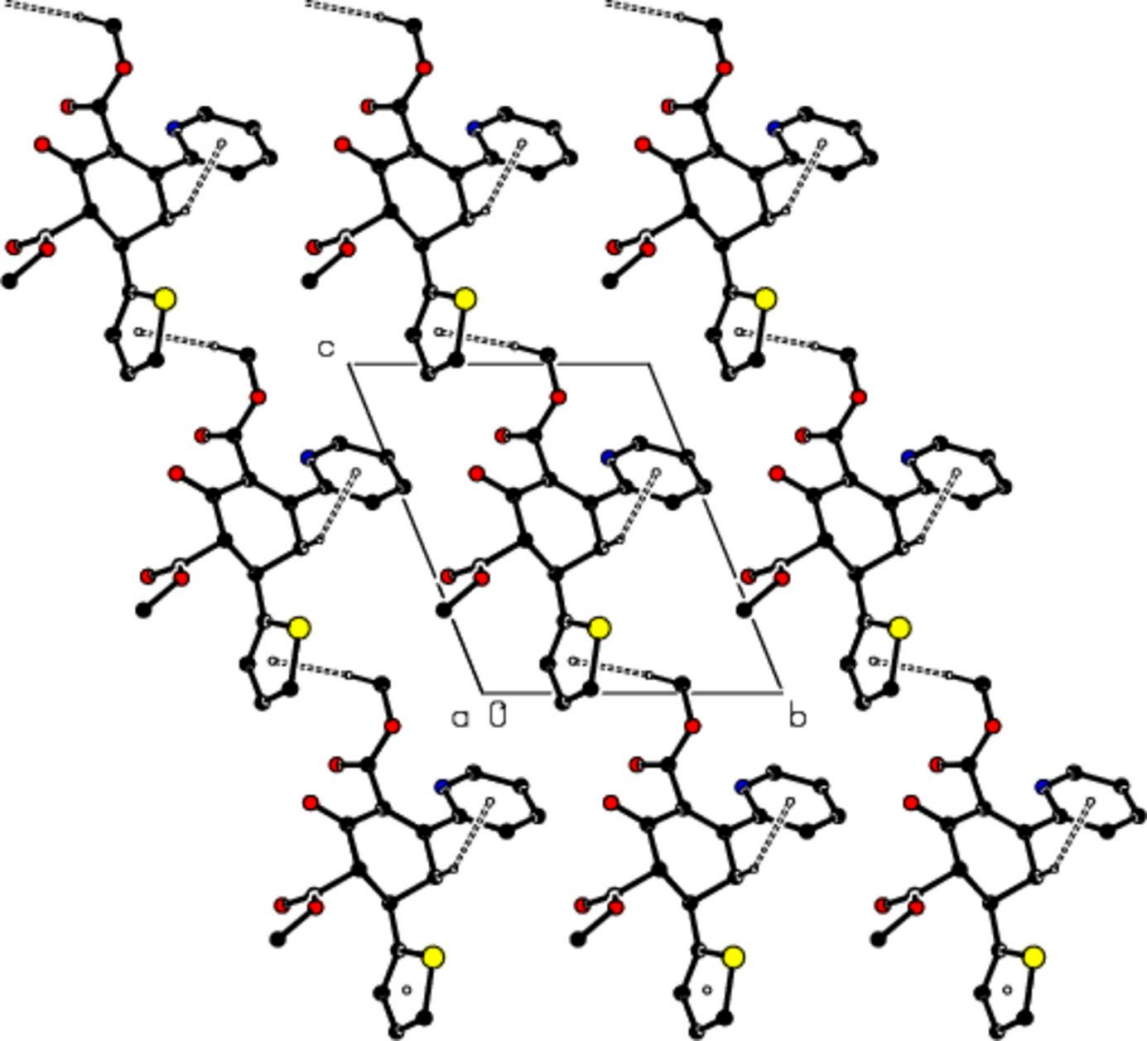
A view of the packing along the *a* axis of the title compound with C—H⋯π inter­actions shown as dashed lines.

In the crystal, mol­ecules are linked by C—H⋯O hydrogen bonds, forming a three-dimensional network (Table 1 ▸; Figs. 2 ▸ and 3 ▸). In addition, C—H⋯π inter­actions connect the mol­ecules, forming layers parallel to the (010) plane, represented by the distances between the same *Cg*1 and the same *Cg*2 centroids (Table 1 ▸; Figs. 4 ▸ and 5 ▸). The lengths of the C—H⋯π inter­actions are similar to the proper hydrogen bonds in the crystal structures. This is reasonable for carbo- and heterocycles (Nishio, 2011 ▸).

**Figure 6:**
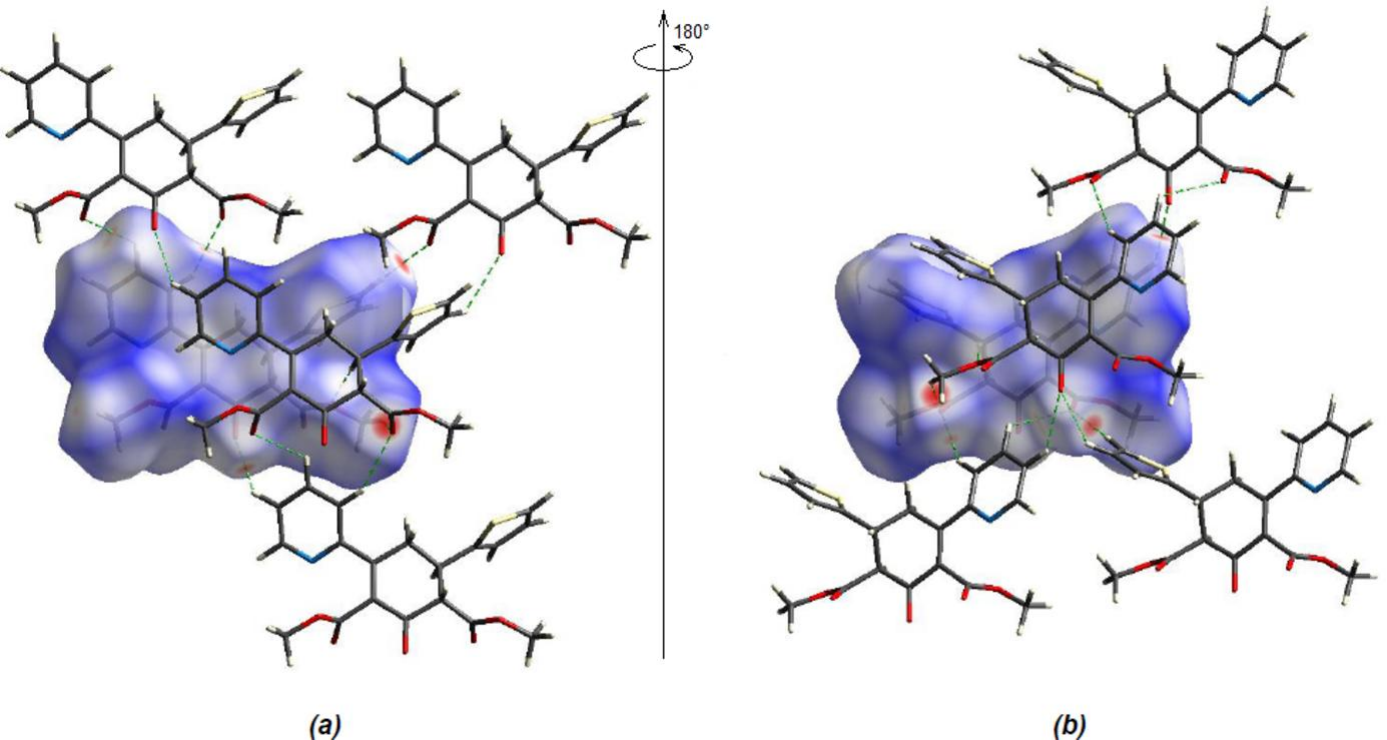
(*a*) Front and (*b*) back sides of the three-dimensional Hirshfeld surface of the title compound mapped over *d_norm_*.

**Figure 7:**
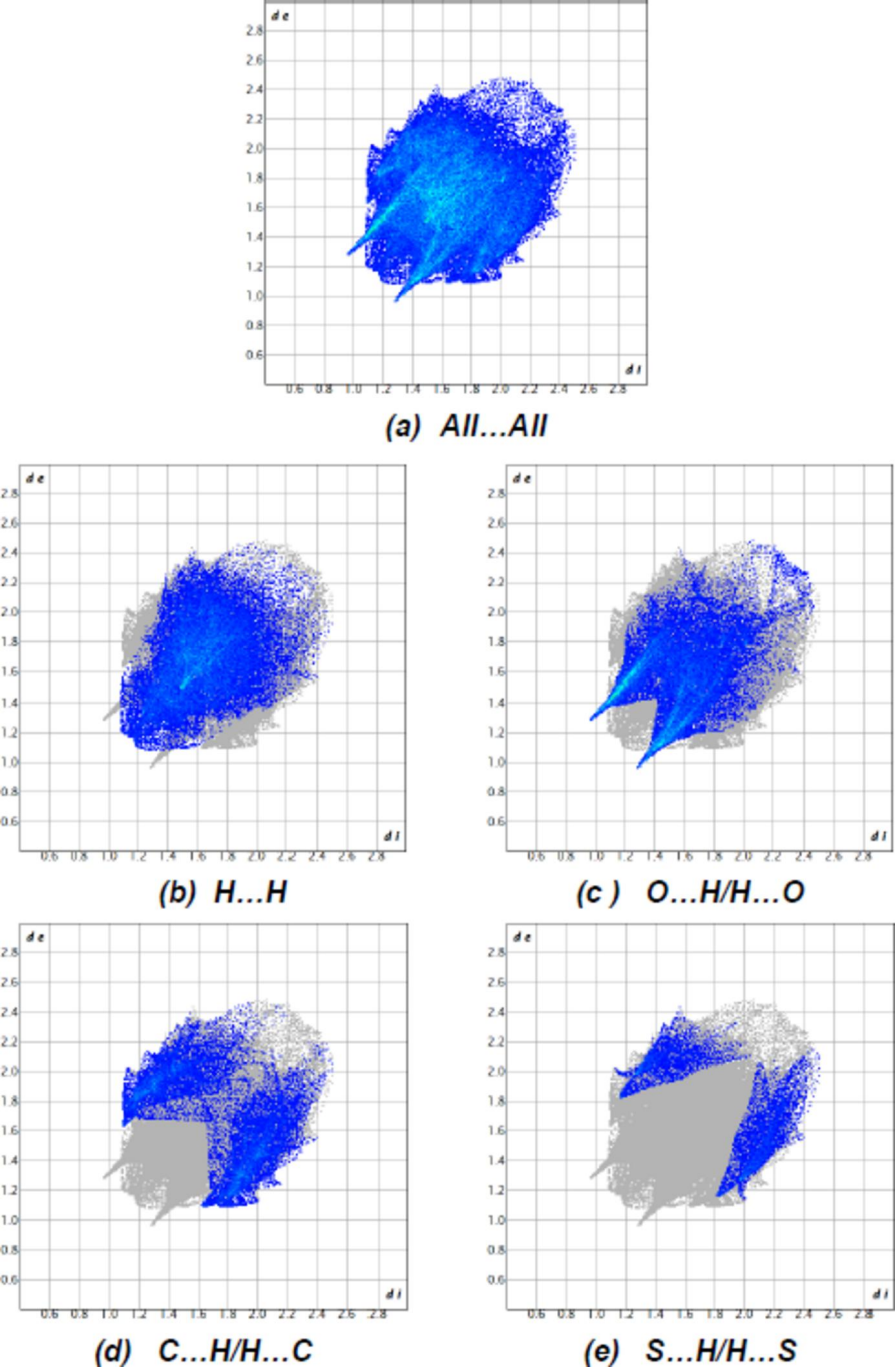
The two-dimensional fingerprint plots, showing (*a*) all inter­actions, and delineated into (*b*) H⋯H, (*c*) O⋯H/H⋯O, (*d*) C⋯H/H⋯C and (*e*) S⋯H/H⋯S inter­actions [*d*_e_ and *d*_i_ represent the distances from a point on the Hirshfeld surface to the nearest atoms outside (external) and inside (inter­nal) the surface, respectively].

*Crystal Explorer 17.5* (Spackman *et al.*, 2021 ▸) was used to generate Hirshfeld surfaces and two-dimensional fingerprint plots in order to qu­antify the inter­molecular inter­actions in the crystal. The Hirshfeld surfaces were mapped over *d*_norm_ in the range −0.2536 (red) to +1.2159 (blue) a.u. (Fig. 6 ▸). The most important inter­atomic contact is H⋯H as it makes the highest contribution to the crystal packing (36.9%, Fig. 7 ▸ *b*). Other major contributors are O⋯H/H⋯O (31.0%, Fig. 7 ▸ *c*), C⋯H/H⋯C (18.9%, Fig. 7 ▸ *d*) and S⋯H/H⋯S (7.9%, Fig. 7 ▸ *e*) inter­actions. Other, smaller contributions are made by N⋯H/H⋯N (2.6%), O⋯O (1.1%), O⋯C/C⋯O (0.9%), N⋯C/C⋯N (0.4%) and C⋯C (0.2%) inter­actions. This distribution is typical for such cyclo­hexene compounds (Naghiyev *et al.*, 2024 ▸).

## 4. Database survey

A search of the Cambridge Structural Database (CSD, Version 5.43, last update November 2022; Groom *et al.*, 2016 ▸) for a central cyclo­hexene or -hexane ring yielded nine compounds related to the title compound, *viz.* CSD refcodes WOMWUU [(**I**); Naghiyev *et al.*, 2024 ▸], UPOMOE [(**II**); Naghiyev *et al.*, 2021*b* ▸], ZOMDUD [(**III**); Gein *et al.*, 2019 ▸], PEWJUZ [(**IV**); Fatahpour *et al.*, 2018 ▸], OZUKAX [(**V**); Tkachenko *et al.*, 2014 ▸], IFUDOD ((**VI**); Gein *et al.*, 2007 ▸], IWEVOV [(**VII**); Mohan *et al.*, 2003 ▸], IWEVUB [(**VIII**); Mohan *et al.*, 2003 ▸] and HALROB [(**IX**); Ravikumar & Mehdi, 1993 ▸].

**Figure 8:**
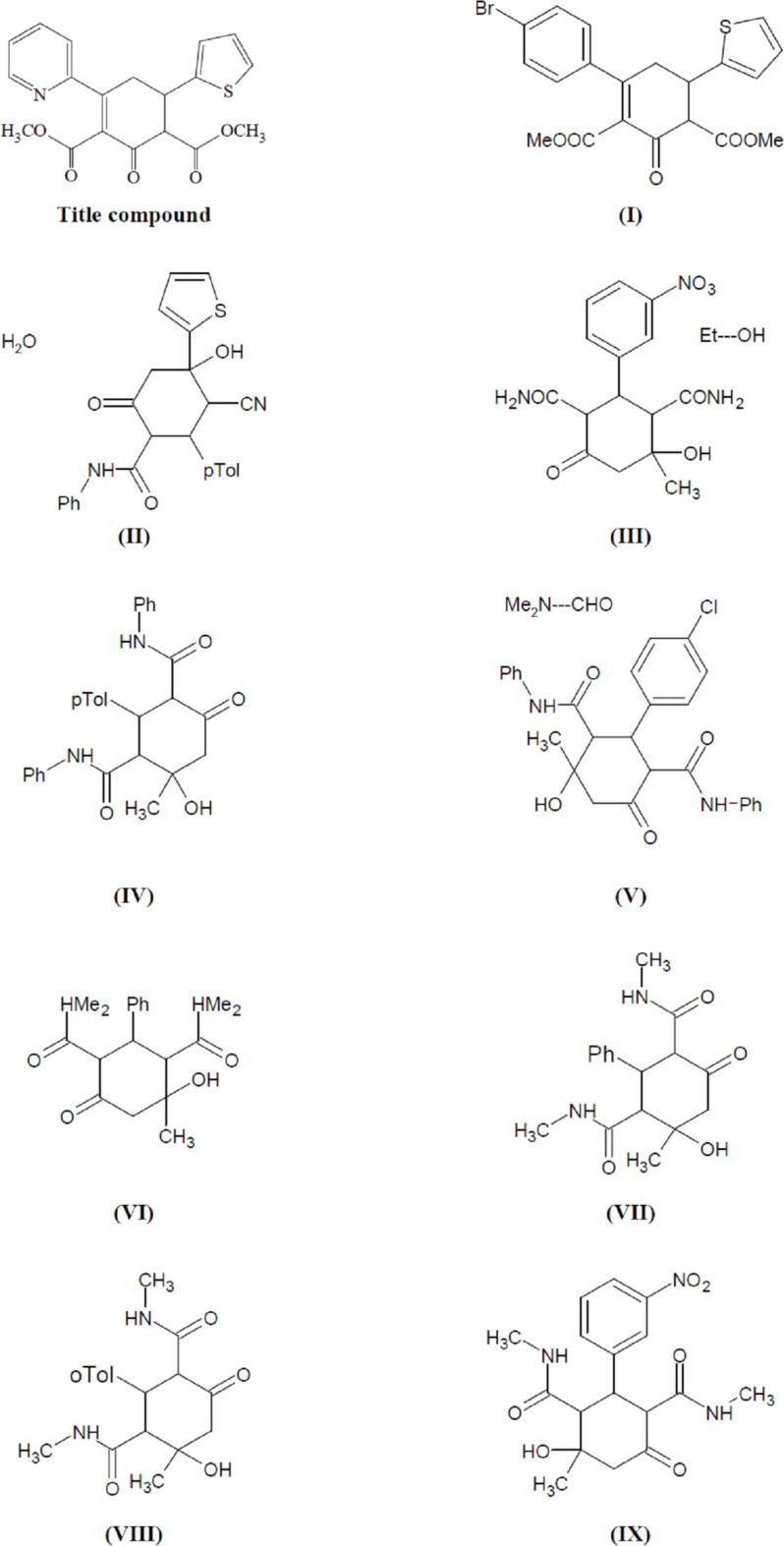
The nine other published cyclo­hexene/hexane-based structures.

Comparing the title compound and previously published structures, the published structures (Fig. 8 ▸) appear to have much higher symmetry space groups. While the title compound crystallizes in the triclinic space group *P*1 with *Z* = 1, (**I**), (**II**) and (**III**) crystallize in the monoclinic space group *P*2_1_/*c*, with *Z* = 4, (**IV**) in *I*2/*c* with *Z* = 4, (**VI**), (**VIII**) and (**IX**) in *P*2_1_/*n* with *Z* = 4, and (**V**) and (**VII**) in the ortho­rhom­bic space group *Pbca* with *Z* = 8.

## 5. Synthesis and crystallization

For a novel synthesis of the title compound, a solution of 1-(pyridin-2-yl)-3-(thio­phen-2-yl)prop-2-en-1-one (7 mmol) and dimethyl-1,3-acetonedi­carboxyl­ate (5.2 mmol) in methanol (30 mL) was stirred for 10 min. Then *N*-methyl­piperazine (3 drops) was added to the reaction mixture, which was stirred for 48 h at room temperature. Then 20 mL of methanol were removed from the reaction mixture, which was left overnight. The precipitated crystals were separated by filtration and recrystallized from an ethanol/water (1:1) solution (m.p. = 480 K, yield 69%).

^1^H NMR (300 MHz, DMSO-*d*_6_, ppm.): 3.30 (*dd*, 2H, CH_2_, *^2^J*_H–H_ = 16.3 and *^3^J*_H–H_ = 8.3); 3.65 (*s*, 6H, 2OCH_3_); 4.02 (*dd*, 1H, CH, *^3^J*_H–H_ = 8.3, *^3^J*_H–H_ = 13.3); 4.20 (*d*, 1H, CH, *^3^J*_H–H_ = 13.3); 7.00 (*t*, 1H, CH_thien._, *^3^J*_H–H_ = 5.1); 7.09 (*d*, 1H, CH_thien._, *^3^J*_H–H_ = 3.5); 7.41 (*d*, 1H, CH_thien._, *^3^J*_H–H_ = 5.1); 7.46 (*t*, 1H, CH_pyrid._, *^3^J*_H–H_ = 7.4); 7.76 (*d*, 1H, CH_pyrid._, *^3^J*_H–H_ = 7.4); 7.91 (*t*, 1H, CH_pyrid._, *^3^J*_H–H_ = 5.7); 8.63 (*d*, 1H, CH_pyrid._, *^3^J*_H–H_ = 5.7). ^13^C NMR (75 MHz, DMSO-*d*_6_, ppm.): 35.47 (CH_2_), 38.23 (CH), 52.24 (OCH_3_), 52.43 (OCH_3_), 60.45 (CH), 123.58 (CH_pyrid._), 125.20 (CH_pyrid._), 125.61 (CH_pyrid._), 125.77 (CH_pyrid._), 127.47 (CH_thien._), 131.43 (C_quat._), 137.93 (CH_thien._), 144.28 (C_thien._), 149.49 (CH_thien._), 153.21 (C_quat._), 155.14 (C_quat._), 166.83 (CO), 169.52 (CO), 191.68 (C=O).

## 6. Refinement

Crystal data, data collection and structure refinement details are summarized in Table 2 ▸. All C-bound H atoms were placed in calculated positions (C—H = 0.95 −1.00 Å) and refined as riding with *U*_iso_(H) = 1.2 or 1.5*U*_eq_(C).

## Supplementary Material

Crystal structure: contains datablock(s) I. DOI: 10.1107/S2056989024004687/oi2007sup1.cif

Structure factors: contains datablock(s) I. DOI: 10.1107/S2056989024004687/oi2007Isup2.hkl

Supporting information file. DOI: 10.1107/S2056989024004687/oi2007Isup3.cml

CCDC reference: 2356669

Additional supporting information:  crystallographic information; 3D view; checkCIF report

## Figures and Tables

**Figure 3:**
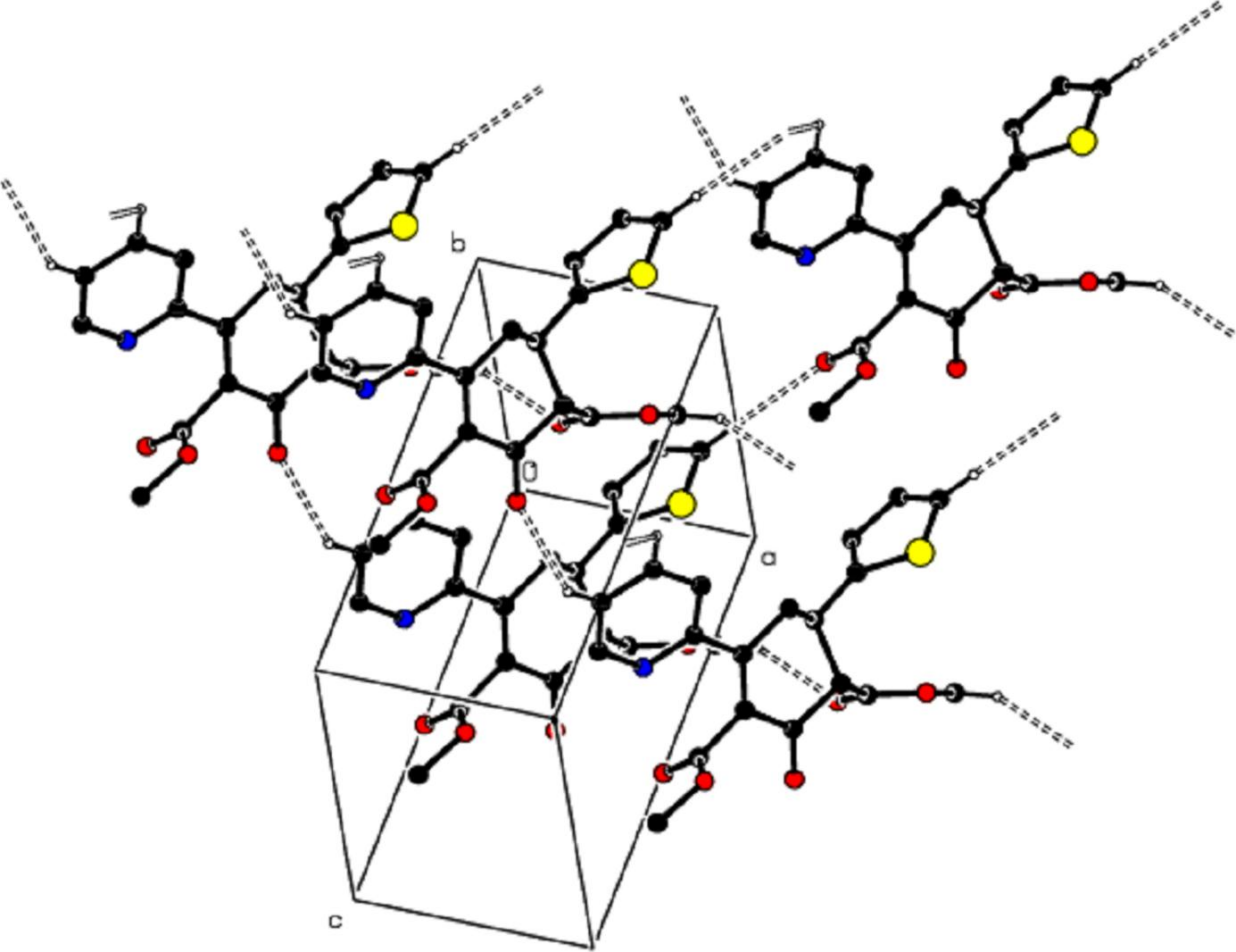
View of partial packing along the *a* axis of the title compound with C—H⋯O hydrogen bonds shown as dashed lines.

**Figure 5:**
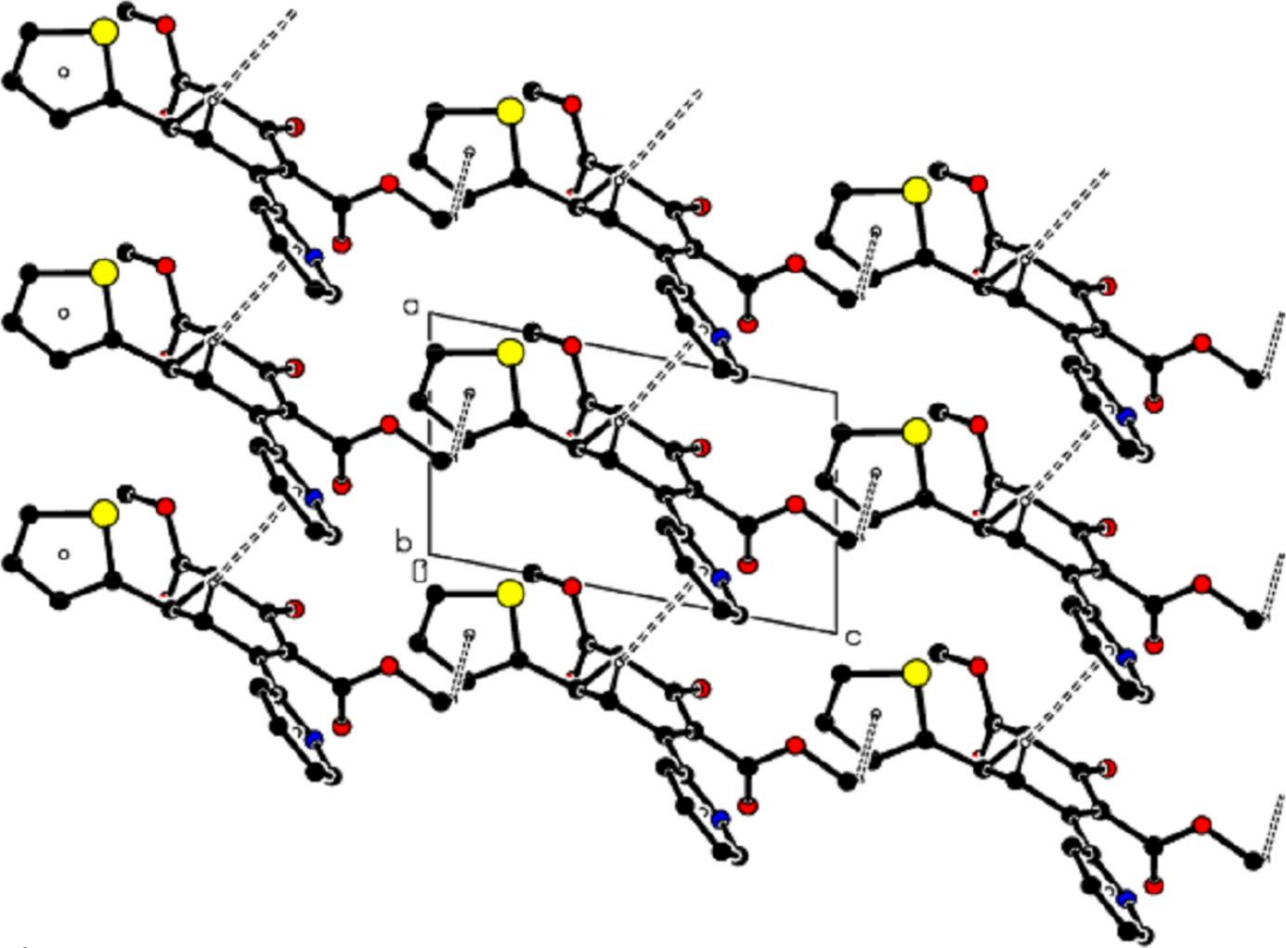
A view of the packing along the *b* axis of the title compound with C—H⋯π inter­actions shown as dashed lines.

**Table 1:** Hydrogen-bond geometry (Å, °) *Cg*1 and *Cg*2 are the centroids of the S1/C9–C12 thio­phene and N1/C13–C17 pyridine rings, respectively.

*D*—H⋯*A*	*D*—H	H⋯*A*	*D*⋯*A*	*D*—H⋯*A*
C3—H3⋯O3^i^	1.00	2.64	3.625 (2)	168
C8—H8*C*⋯O2^ii^	0.98	2.35	3.215 (3)	146
C11—H11⋯O1^iii^	0.95	2.62	3.193 (2)	119
C12—H12⋯O4^iv^	0.95	2.50	3.446 (2)	180
C14—H14⋯O2^v^	0.95	2.61	3.468 (2)	151
C16—H16⋯O1^vi^	0.95	2.52	3.305 (3)	140
C19—H19*B*⋯O2^vii^	0.98	2.64	3.584 (3)	161
C4—H4*A*⋯*Cg*2^ii^	0.99	2.94	3.841 (2)	152
C19—H19*C*⋯*Cg*1^viii^	0.98	2.78	3.659 (3)	149

Symmetry codes: (i) 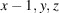 ; (ii) 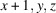 ; (iii) 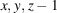 ; (iv) 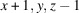 ; (v) 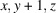 ; (vi) 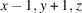 ; (vii) 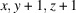 ; (viii) 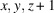 .

**Table 2:** Experimental details

Crystal data	
Chemical formula	C_19_H_17_NO_5_S
*M*_r_	371.39
Crystal system, space group	Triclinic, *P*1
Temperature (K)	100
*a*, *b*, *c* (Å)	5.5260 (1), 8.5012 (1), 10.1076 (2)
α, β, γ (°)	110.910 (2), 98.128 (1), 96.006 (1)
*V* (Å^3^)	432.88 (1)
*Z*	1
Radiation type	Cu *K*α
μ (mm^−1^)	1.94
Crystal size (mm)	0.25 × 0.23 × 0.09

Data collection	
Diffractometer	XtaLAB Synergy, Dualflex, HyPix
Absorption correction	Gaussian (*CrysAlis PRO*; Rigaku OD, 2022 ▸)
*T*_min_, *T*_max_	0.647, 0.840
No. of measured, independent and observed [*I* > 2σ(*I*)] reflections	18199, 3530, 3524
*R*_int_	0.023
(sin θ/λ)_max_ (Å^−1^)	0.634

Refinement	
*R*[*F* ^2^ > 2σ(*F* ^2^)], *wR*(*F* ^2^), *S*	0.023, 0.059, 1.06
No. of reflections	3530
No. of parameters	238
No. of restraints	3
H-atom treatment	H-atom parameters constrained
Δρ_max_, Δρ_min_ (e Å^−3^)	0.22, −0.18
Absolute structure	Flack *x* determined using 1675 quotients [(*I* ^+^)−(*I* ^−^)]/[(*I* ^+^)+(*I* ^−^)] (Parsons *et al.*, 2013 ▸)
Absolute structure parameter	0.003 (7)

Computer programs: *CrysAlis PRO* (Rigaku OD, 2022 ▸), *SHELXT2014/5* (Sheldrick, 2015*a* ▸), *SHELXL2019/3* (Sheldrick, 2015*b* ▸), *ORTEP-3 for Windows* (Farrugia, 2012 ▸) and *PLATON* (Spek, 2020 ▸).

## supplementary crystallographic information

### Crystal data

**Table:**

C_19_H_17_NO_5_S	*Z* = 1
*M**_r_* = 371.39	*F*(000) = 194
Triclinic, *P*1	*D*_x_ = 1.425 Mg m^−^^3^
*a* = 5.5260 (1) Å	Cu *K*α radiation, λ = 1.54184 Å
*b* = 8.5012 (1) Å	Cell parameters from 16321 reflections
*c* = 10.1076 (2) Å	θ = 4.8–77.4°
α = 110.910 (2)°	µ = 1.94 mm^−^^1^
β = 98.128 (1)°	*T* = 100 K
γ = 96.006 (1)°	Prism, colourless
*V* = 432.88 (1) Å^3^	0.25 × 0.23 × 0.09 mm

### Data collection

**Table:**

XtaLAB Synergy, Dualflex, HyPix diffractometer	3524 reflections with *I* > 2σ(*I*)
Radiation source: micro-focus sealed X-ray tube	*R*_int_ = 0.023
φ and ω scans	θ_max_ = 77.8°, θ_min_ = 4.8°
Absorption correction: gaussian (CrysAlisPro; Rigaku OD, 2022)	*h* = −6→6
*T*_min_ = 0.647, *T*_max_ = 0.840	*k* = −10→10
18199 measured reflections	*l* = −12→12
3530 independent reflections	

### Refinement

**Table:**

Refinement on *F*^2^	H-atom parameters constrained
Least-squares matrix: full	*w* = 1/[σ^2^(*F*_o_^2^) + (0.0337*P*)^2^ + 0.097*P*] where *P* = (*F*_o_^2^ + 2*F*_c_^2^)/3
*R*[*F*^2^ > 2σ(*F*^2^)] = 0.023	(Δ/σ)_max_ < 0.001
*wR*(*F*^2^) = 0.059	Δρ_max_ = 0.22 e Å^−^^3^
*S* = 1.06	Δρ_min_ = −0.17 e Å^−^^3^
3530 reflections	Extinction correction: *SHELXL2019/2* (Sheldrick, 2015b), Fc^*^=kFc[1+0.001xFc^2^λ^3^/sin(2θ)]^-1/4^
238 parameters	Extinction coefficient: 0.0131 (14)
3 restraints	Absolute structure: Flack *x* determined using 1675 quotients [(*I*^+^)-(*I*^-^)]/[(*I*^+^)+(*I*^-^)] (Parsons *et al.*, 2013)
Hydrogen site location: inferred from neighbouring sites	Absolute structure parameter: 0.003 (7)

### Special details

**Table:**

Geometry. All esds (except the esd in the dihedral angle between two l.s. planes) are estimated using the full covariance matrix. The cell esds are taken into account individually in the estimation of esds in distances, angles and torsion angles; correlations between esds in cell parameters are only used when they are defined by crystal symmetry. An approximate (isotropic) treatment of cell esds is used for estimating esds involving l.s. planes.

### Fractional atomic coordinates and isotropic or equivalent isotropic displacement parameters (Å^2^)

**Table:**

	*x*	*y*	*z*	*U*_iso_*/*U*_eq_	
C1	0.6259 (4)	0.3716 (2)	0.6011 (2)	0.0132 (4)	
C2	0.7320 (4)	0.3495 (2)	0.46536 (19)	0.0127 (4)	
H2	0.898999	0.422837	0.493270	0.015*	
C3	0.5592 (4)	0.4059 (2)	0.36259 (19)	0.0136 (4)	
H3	0.390686	0.337398	0.343691	0.016*	
C4	0.5382 (4)	0.5930 (2)	0.4416 (2)	0.0146 (4)	
H4A	0.703452	0.664844	0.465810	0.017*	
H4B	0.425908	0.630379	0.377470	0.017*	
C5	0.4396 (3)	0.6170 (2)	0.5780 (2)	0.0134 (4)	
C6	0.4809 (4)	0.5121 (2)	0.64983 (19)	0.0129 (4)	
C7	0.7580 (4)	0.1653 (2)	0.3869 (2)	0.0142 (4)	
C8	1.0028 (4)	−0.0161 (3)	0.2524 (3)	0.0281 (5)	
H8A	0.894696	−0.044480	0.158603	0.042*	
H8B	0.954145	−0.100288	0.293541	0.042*	
H8C	1.175204	−0.017674	0.239795	0.042*	
C9	0.6332 (4)	0.3713 (2)	0.2190 (2)	0.0144 (4)	
C10	0.5052 (4)	0.2567 (3)	0.0887 (2)	0.0188 (4)	
H10	0.352786	0.185818	0.077251	0.023*	
C11	0.6230 (4)	0.2541 (3)	−0.0286 (2)	0.0211 (4)	
H11	0.557058	0.181613	−0.126180	0.025*	
C12	0.8386 (4)	0.3654 (3)	0.0135 (2)	0.0198 (4)	
H12	0.941797	0.380424	−0.050162	0.024*	
C13	0.2915 (4)	0.7557 (2)	0.6266 (2)	0.0138 (4)	
C14	0.3097 (4)	0.8926 (2)	0.5803 (2)	0.0170 (4)	
H14	0.419867	0.900060	0.517866	0.020*	
C15	0.1640 (4)	1.0168 (2)	0.6273 (2)	0.0193 (4)	
H15	0.173481	1.111309	0.597847	0.023*	
C16	0.0042 (4)	1.0017 (3)	0.7177 (2)	0.0192 (4)	
H16	−0.098664	1.084695	0.750789	0.023*	
C17	−0.0018 (4)	0.8619 (3)	0.7589 (2)	0.0182 (4)	
H17	−0.109760	0.852595	0.822018	0.022*	
C18	0.3784 (4)	0.5217 (2)	0.7826 (2)	0.0132 (4)	
C19	0.3964 (4)	0.6765 (3)	1.0269 (2)	0.0222 (4)	
H19A	0.220812	0.685154	1.005687	0.033*	
H19B	0.484406	0.781046	1.106272	0.033*	
H19C	0.411825	0.578189	1.054703	0.033*	
N1	0.1361 (3)	0.7402 (2)	0.71452 (18)	0.0161 (3)	
O1	0.6615 (3)	0.27874 (17)	0.66693 (15)	0.0178 (3)	
O2	0.5944 (3)	0.04488 (18)	0.35502 (15)	0.0196 (3)	
O3	0.9794 (3)	0.15282 (18)	0.34916 (15)	0.0188 (3)	
O4	0.2142 (3)	0.41766 (17)	0.78248 (15)	0.0175 (3)	
O5	0.5031 (3)	0.65445 (18)	0.89989 (14)	0.0168 (3)	
S1	0.90193 (7)	0.47674 (5)	0.19781 (5)	0.01854 (13)	

### Atomic displacement parameters (Å^2^)

**Table:**

	*U*^11^	*U*^22^	*U*^33^	*U*^12^	*U*^13^	*U*^23^
C1	0.0118 (9)	0.0139 (8)	0.0134 (8)	0.0017 (7)	0.0025 (7)	0.0047 (7)
C2	0.0131 (9)	0.0131 (8)	0.0128 (8)	0.0041 (7)	0.0039 (7)	0.0049 (7)
C3	0.0140 (9)	0.0149 (9)	0.0123 (8)	0.0022 (7)	0.0033 (7)	0.0056 (7)
C4	0.0166 (10)	0.0143 (8)	0.0152 (9)	0.0053 (7)	0.0044 (7)	0.0072 (7)
C5	0.0122 (9)	0.0125 (8)	0.0144 (8)	0.0016 (7)	0.0026 (7)	0.0039 (7)
C6	0.0133 (9)	0.0129 (8)	0.0125 (9)	0.0028 (7)	0.0048 (7)	0.0039 (7)
C7	0.0157 (10)	0.0173 (9)	0.0114 (8)	0.0058 (7)	0.0039 (7)	0.0065 (7)
C8	0.0193 (11)	0.0212 (11)	0.0342 (12)	0.0067 (8)	0.0092 (9)	−0.0032 (9)
C9	0.0153 (10)	0.0160 (8)	0.0149 (9)	0.0054 (7)	0.0059 (7)	0.0075 (7)
C10	0.0185 (10)	0.0228 (10)	0.0153 (9)	0.0030 (8)	0.0048 (8)	0.0071 (8)
C11	0.0245 (11)	0.0256 (10)	0.0137 (9)	0.0051 (9)	0.0059 (8)	0.0071 (8)
C12	0.0226 (11)	0.0253 (10)	0.0147 (9)	0.0066 (8)	0.0083 (8)	0.0088 (8)
C13	0.0136 (10)	0.0125 (8)	0.0137 (8)	0.0024 (7)	0.0019 (7)	0.0033 (7)
C14	0.0189 (11)	0.0157 (9)	0.0179 (9)	0.0036 (8)	0.0040 (8)	0.0078 (8)
C15	0.0211 (11)	0.0135 (9)	0.0223 (10)	0.0032 (8)	0.0002 (8)	0.0067 (8)
C16	0.0182 (10)	0.0152 (9)	0.0204 (9)	0.0068 (7)	0.0016 (8)	0.0019 (7)
C17	0.0167 (10)	0.0201 (9)	0.0173 (9)	0.0064 (8)	0.0054 (7)	0.0046 (8)
C18	0.0166 (10)	0.0119 (8)	0.0129 (8)	0.0061 (7)	0.0048 (7)	0.0049 (7)
C19	0.0245 (11)	0.0264 (10)	0.0127 (9)	0.0052 (9)	0.0070 (8)	0.0023 (8)
N1	0.0166 (9)	0.0159 (8)	0.0165 (8)	0.0055 (6)	0.0050 (6)	0.0057 (6)
O1	0.0222 (8)	0.0200 (7)	0.0170 (6)	0.0095 (6)	0.0071 (6)	0.0107 (5)
O2	0.0192 (8)	0.0158 (7)	0.0218 (7)	0.0010 (6)	0.0061 (6)	0.0045 (5)
O3	0.0150 (7)	0.0166 (7)	0.0222 (7)	0.0055 (5)	0.0065 (6)	0.0023 (6)
O4	0.0219 (8)	0.0150 (6)	0.0170 (6)	0.0035 (6)	0.0083 (6)	0.0059 (5)
O5	0.0183 (7)	0.0181 (7)	0.0128 (6)	0.0020 (5)	0.0049 (5)	0.0039 (5)
S1	0.0179 (2)	0.0223 (2)	0.0148 (2)	−0.00028 (18)	0.00576 (16)	0.00623 (17)

### Geometric parameters (Å, º)

**Table:**

C1—O1	1.215 (2)	C10—C11	1.426 (3)
C1—C6	1.484 (3)	C10—H10	0.9500
C1—C2	1.526 (2)	C11—C12	1.353 (3)
C2—C7	1.515 (3)	C11—H11	0.9500
C2—C3	1.546 (3)	C12—S1	1.725 (2)
C2—H2	1.0000	C12—H12	0.9500
C3—C9	1.499 (2)	C13—N1	1.349 (2)
C3—C4	1.529 (2)	C13—C14	1.400 (3)
C3—H3	1.0000	C14—C15	1.384 (3)
C4—C5	1.509 (3)	C14—H14	0.9500
C4—H4A	0.9900	C15—C16	1.386 (3)
C4—H4B	0.9900	C15—H15	0.9500
C5—C6	1.353 (3)	C16—C17	1.391 (3)
C5—C13	1.485 (3)	C16—H16	0.9500
C6—C18	1.508 (2)	C17—N1	1.335 (3)
C7—O2	1.207 (2)	C17—H17	0.9500
C7—O3	1.335 (2)	C18—O4	1.199 (2)
C8—O3	1.452 (2)	C18—O5	1.344 (2)
C8—H8A	0.9800	C19—O5	1.448 (2)
C8—H8B	0.9800	C19—H19A	0.9800
C8—H8C	0.9800	C19—H19B	0.9800
C9—C10	1.364 (3)	C19—H19C	0.9800
C9—S1	1.733 (2)		
			
O1—C1—C6	121.66 (16)	C3—C9—S1	123.13 (14)
O1—C1—C2	121.05 (17)	C9—C10—C11	113.26 (19)
C6—C1—C2	117.28 (15)	C9—C10—H10	123.4
C7—C2—C1	111.28 (15)	C11—C10—H10	123.4
C7—C2—C3	108.90 (15)	C12—C11—C10	112.96 (18)
C1—C2—C3	109.64 (15)	C12—C11—H11	123.5
C7—C2—H2	109.0	C10—C11—H11	123.5
C1—C2—H2	109.0	C11—C12—S1	111.23 (15)
C3—C2—H2	109.0	C11—C12—H12	124.4
C9—C3—C4	113.33 (15)	S1—C12—H12	124.4
C9—C3—C2	113.64 (16)	N1—C13—C14	122.33 (18)
C4—C3—C2	108.56 (15)	N1—C13—C5	116.34 (17)
C9—C3—H3	107.0	C14—C13—C5	121.32 (17)
C4—C3—H3	107.0	C15—C14—C13	118.75 (18)
C2—C3—H3	107.0	C15—C14—H14	120.6
C5—C4—C3	110.93 (15)	C13—C14—H14	120.6
C5—C4—H4A	109.5	C14—C15—C16	119.19 (19)
C3—C4—H4A	109.5	C14—C15—H15	120.4
C5—C4—H4B	109.5	C16—C15—H15	120.4
C3—C4—H4B	109.5	C15—C16—C17	118.38 (19)
H4A—C4—H4B	108.0	C15—C16—H16	120.8
C6—C5—C13	122.14 (17)	C17—C16—H16	120.8
C6—C5—C4	120.52 (17)	N1—C17—C16	123.48 (19)
C13—C5—C4	117.32 (16)	N1—C17—H17	118.3
C5—C6—C1	122.52 (16)	C16—C17—H17	118.3
C5—C6—C18	123.87 (17)	O4—C18—O5	125.11 (17)
C1—C6—C18	113.58 (15)	O4—C18—C6	123.28 (18)
O2—C7—O3	123.86 (18)	O5—C18—C6	111.52 (16)
O2—C7—C2	124.32 (18)	O5—C19—H19A	109.5
O3—C7—C2	111.70 (16)	O5—C19—H19B	109.5
O3—C8—H8A	109.5	H19A—C19—H19B	109.5
O3—C8—H8B	109.5	O5—C19—H19C	109.5
H8A—C8—H8B	109.5	H19A—C19—H19C	109.5
O3—C8—H8C	109.5	H19B—C19—H19C	109.5
H8A—C8—H8C	109.5	C17—N1—C13	117.88 (17)
H8B—C8—H8C	109.5	C7—O3—C8	114.76 (16)
C10—C9—C3	126.53 (18)	C18—O5—C19	113.04 (15)
C10—C9—S1	110.34 (15)	C12—S1—C9	92.21 (10)
			
O1—C1—C2—C7	29.2 (3)	C3—C9—C10—C11	179.62 (19)
C6—C1—C2—C7	−151.60 (17)	S1—C9—C10—C11	−0.2 (2)
O1—C1—C2—C3	149.70 (18)	C9—C10—C11—C12	0.2 (3)
C6—C1—C2—C3	−31.1 (2)	C10—C11—C12—S1	−0.2 (2)
C7—C2—C3—C9	−51.8 (2)	C6—C5—C13—N1	−20.6 (3)
C1—C2—C3—C9	−173.75 (15)	C4—C5—C13—N1	157.70 (17)
C7—C2—C3—C4	−178.86 (15)	C6—C5—C13—C14	160.74 (19)
C1—C2—C3—C4	59.17 (19)	C4—C5—C13—C14	−20.9 (3)
C9—C3—C4—C5	174.49 (16)	N1—C13—C14—C15	0.4 (3)
C2—C3—C4—C5	−58.3 (2)	C5—C13—C14—C15	178.96 (17)
C3—C4—C5—C6	28.6 (3)	C13—C14—C15—C16	−0.3 (3)
C3—C4—C5—C13	−149.71 (17)	C14—C15—C16—C17	0.5 (3)
C13—C5—C6—C1	179.18 (17)	C15—C16—C17—N1	−0.9 (3)
C4—C5—C6—C1	0.9 (3)	C5—C6—C18—O4	107.7 (2)
C13—C5—C6—C18	1.4 (3)	C1—C6—C18—O4	−70.2 (2)
C4—C5—C6—C18	−176.85 (18)	C5—C6—C18—O5	−75.7 (2)
O1—C1—C6—C5	179.91 (19)	C1—C6—C18—O5	106.35 (19)
C2—C1—C6—C5	0.7 (3)	C16—C17—N1—C13	0.9 (3)
O1—C1—C6—C18	−2.1 (3)	C14—C13—N1—C17	−0.7 (3)
C2—C1—C6—C18	178.65 (16)	C5—C13—N1—C17	−179.31 (17)
C1—C2—C7—O2	50.6 (3)	O2—C7—O3—C8	6.2 (3)
C3—C2—C7—O2	−70.3 (2)	C2—C7—O3—C8	−169.87 (18)
C1—C2—C7—O3	−133.27 (17)	O4—C18—O5—C19	−8.5 (3)
C3—C2—C7—O3	105.76 (18)	C6—C18—O5—C19	174.97 (15)
C4—C3—C9—C10	−122.9 (2)	C11—C12—S1—C9	0.08 (18)
C2—C3—C9—C10	112.5 (2)	C10—C9—S1—C12	0.06 (16)
C4—C3—C9—S1	56.8 (2)	C3—C9—S1—C12	−179.74 (17)
C2—C3—C9—S1	−67.7 (2)		

### Hydrogen-bond geometry (Å, º)

*Cg*1 and *Cg*2 are the centroids of the S1/C9–C12 thiophene and N1/C13–C17 pyridine rings, respectively.

**Table:**

*D*—H···*A*	*D*—H	H···*A*	*D*···*A*	*D*—H···*A*
C3—H3···O3^i^	1.00	2.64	3.625 (2)	168
C8—H8*C*···O2^ii^	0.98	2.35	3.215 (3)	146
C11—H11···O1^iii^	0.95	2.62	3.193 (2)	119
C12—H12···O4^iv^	0.95	2.50	3.446 (2)	180
C14—H14···O2^v^	0.95	2.61	3.468 (2)	151
C16—H16···O1^vi^	0.95	2.52	3.305 (3)	140
C19—H19*B*···O2^vii^	0.98	2.64	3.584 (3)	161
C4—H4*A*···*Cg*2^ii^	0.99	2.94	3.841 (2)	152
C19—H19*C*···*Cg*1^viii^	0.98	2.78	3.659 (3)	149

Symmetry codes: (i) *x*−1, *y*, *z*; (ii) *x*+1, *y*, *z*; (iii) *x*, *y*, *z*−1; (iv) *x*+1, *y*, *z*−1; (v) *x*, *y*+1, *z*; (vi) *x*−1, *y*+1, *z*; (vii) *x*, *y*+1, *z*+1; (viii) *x*, *y*, *z*+1.
